# Weakly supervised learning for classification of lung cytological images using attention-based multiple instance learning

**DOI:** 10.1038/s41598-021-99246-4

**Published:** 2021-10-13

**Authors:** Atsushi Teramoto, Yuka Kiriyama, Tetsuya Tsukamoto, Eiko Sakurai, Ayano Michiba, Kazuyoshi Imaizumi, Kuniaki Saito, Hiroshi Fujita

**Affiliations:** 1 School of Medical Sciences, Fujita Health University, Aichi, Japan; 2 School of Medicine, Fujita Health University, Aichi, Japan; 3 Graduate School of Medicine, Fujita Health University, Aichi, Japan; 4 Faculty of Engineering, Gifu University, Gifu, Japan

**Keywords:** Information technology, Machine learning, Lung cancer

## Abstract

In cytological examination, suspicious cells are evaluated regarding malignancy and cancer type. To assist this, we previously proposed an automated method based on supervised learning that classifies cells in lung cytological images as benign or malignant. However, it is often difficult to label all cells. In this study, we developed a weakly supervised method for the classification of benign and malignant lung cells in cytological images using attention-based deep multiple instance learning (AD MIL). Images of lung cytological specimens were divided into small patch images and stored in bags. Each bag was then labeled as benign or malignant, and classification was conducted using AD MIL. The distribution of attention weights was also calculated as a color map to confirm the presence of malignant cells in the image. AD MIL using the AlexNet-like convolutional neural network model showed the best classification performance, with an accuracy of 0.916, which was better than that of supervised learning. In addition, an attention map of the entire image based on the attention weight allowed AD MIL to focus on most malignant cells. Our weakly supervised method automatically classifies cytological images with acceptable accuracy based on supervised learning without complex annotations.

## Introduction

Lung cancer is the leading cause of cancer death among males and females worldwide, and early and accurate diagnosis is critical for survival^1^. When the presence of a tumor is noted in a chest X-ray or CT examination, pathological examination is performed to classify it as benign or malignant^2^. Pathological examination includes cytology and histology. In cytology, a cytotechnologist (screener) first screens the samples and finds suspicious cells. The cytopathologist then observes the identified cells in detail and a decision is sometimes made using a combination of regular cytology specimens and immunostaining specimens. Cytology, the first step in detecting malignant cells, plays an important role in influencing the final diagnostic results. However, the screener must find suspicious cells within a large number of cells. The task is extremely burdensome, and there are concerns about the variation in diagnostic accuracy among screeners. Therefore, in this study, we focused on the classification of benign and malignant lung cells based on deep learning technology, which has excellent image recognition capability.

Deep learning technology is an application of multilayer neural network technology and convolutional neural networks (CNNs), which were developed with inspiration from the workings of vision, and is widely used for image classification, object detection, and prediction. There are also many applications in medical imaging^3–9^. For pathological images, CNN has also been used to determine the distribution of malignant cells^10,11^ and to classify cells^12,13^. We have developed diagnostic support technology for lung cytology, and have proposed methods that use CNN to classify cells as benign or malignant^14^.

In these studies, target image and label (correct answer) pairs for that image are given to the deep learning model for training, which is called supervised learning. Good classification performance is achieved when the correct label is assigned to each image. On the contrary, if the labels are inaccurate, the classification performance will be degraded. In cytology, there are some similarities between benign and malignant cells, and there are also many atypical cells. Therefore, it is difficult to assign accurate labels of benign and malignant to individual cells. Recently, multiple instance learning (MIL) has been attracting attention as a weakly supervised learning method that can train networks without creating labels on a one-to-one basis^15^.

MIL introduces the concept of “instances” that represent individual data and “bags” that contain instances that belong to groups such as cases. The network performs the classification in bag units. In this case, even if there are extra or incorrect instances in the bag that do not contribute to the classification, their influence is low because the classification is performed comprehensively on a bag-by-bag basis. Recently, several methods that introduce attention mechanisms and deep learning^16^ have been proposed^17,18^. An attention mechanism is a technique to evaluate which information should be paid attention to when a target contains a large amount of information. Recently, it has been widely used in the fields of natural language processing and image recognition. By clarifying the areas to be focused on, better performance can be obtained compared to that obtained using conventional methods. By visualizing the internal parameters of the attention mechanism, it is possible to determine the instances that have been paid attention to, and to visualize the basis for the decision. Deep learning techniques are also effective in extracting features from instances and processing attention mechanisms. Ilse et al. proposed an attention-based deep MIL (AD MIL) using deep learning and the attention mechanism, and showed that it can be applied to text and cell classifications in histological images^18^. In addition, Hashimoto et al. applied MIL processing of whole slide images at multiple scales to classify lymphoma subtypes^17^.

To the best of our knowledge, there are no studies on the classification of benign and malignant cells in lung cytological images using weakly supervised learning, and no comparison has been made with supervised learning. In this study, we developed a method for the classification of benign and malignant cytological images using AD MIL and compared its performance with that of supervised learning.

## Methods

### Outline of proposed scheme

**Figure 1:**
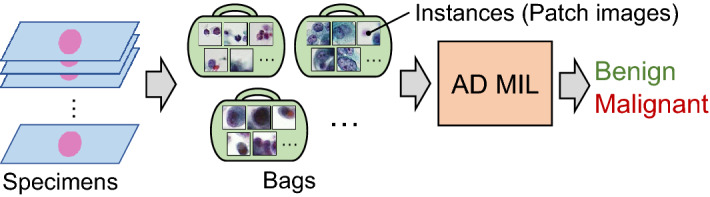
Schematic﻿ diagram of the proposed method.

An outline of the proposed method is shown in Fig. 1. Images of lung cytological specimens were divided into small patch images and stored in bags with case IDs. Each bag was then labeled as benign or malignant, and the classification of benign or malignant was performed by supplying the bag units to the AD MIL.

### Image dataset

This study was performed as a retrospective study with permission from the Institutional Review Board of Fujita Health University (IRB number: HM16-155). The informed consent was obtained from patients subject to the condition of data anonymization. All experimental protocols were performed in accordance with the relevant guidelines and regulations in compliance with the Declaration of Helsinki. For this study, lung cells of 322 patients were collected with interventional cytology using either bronchoscopy or CT-guided fine-needle aspiration cytology, and comprised 108 benign and 214 malignant cases. Malignant cases comprised 124 adenocarcinomas, 52 squamous cell carcinomas, and 38 small cell carcinomas. In these diagnoses, a final decision was made in conjunction with the histological analysis of specimen from a biopsy. Biopsy tissues were collected simultaneously with cytological specimens, fixed in 10% formalin, dehydrated, and embedded in paraffin. In some cases, where diagnosis was difficult, the 3 μm tissue sections were subjected to immunohistochemical analysis to make the decision. Cytological specimens were prepared with liquid-based cytology using the BD SurePathTM liquid-based Pap test (Beckton Dickinson, Franklin Lakes, NJ, USA) and were stained using the Papanicolaou method. Using a digital camera (DP20, Olympus Corporation, Tokyo, Japan) attached to a microscope (BX53, Olympus Corporation) with a 40 × objective lens, 1252 microscopic images of benign cells and 1805 of malignant cells were acquired in a JPEG format with a size of 1280 × 960 pixels per image.

To classify these images, it is important to understand the characteristics of the cells of interest. The characteristics of benign and malignant cells are described below.

#### Characteristics of benign cells

Benign cells have smaller, more uniform nuclei, and smoother cell borders. Nucleoli are small and few. The volume of cytoplasm is large, and normal pulmonary bronchial epithelial cells have cilia.

#### Characteristics of malignant cells

The characteristics of malignant cells vary greatly, depending on their tissue type (adenocarcinoma, squamous cell carcinoma, small cell carcinoma). In general, malignant cells have less cytoplasm and irregularly shaped nuclei. In adenocarcinoma, large nucleoli are often observed and the chromatin particles are rough. In addition, nuclei are often unevenly distributed in the cytoplasm. Squamous cell carcinoma, which is often confused with adenocarcinoma, has smaller nucleoli and finer chromatin particles. The cytoplasm is thick, and the nuclei are often located near the center of the cytoplasm. In these two histological types, the nuclei are larger than in normal cells, whereas in small cell carcinoma, the nuclei are smaller and are often interconnected. On Papanicolaou staining, the nuclei of small cell carcinoma cells are darker than those of other histological types and appear different in color from other cells.

### Generation of instances

**Figure 2:**
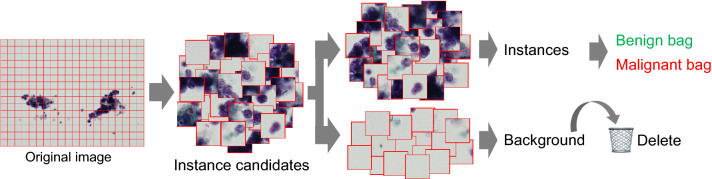
Generation﻿ of instances for benign and malignant bags.

Because MIL can classify a batch of data based on a large amount of data, each specimen image with a matrix size of 1280 × 960 was divided into small patches that were used as instance candidates (Fig. 2). For the matrix size of patch images, we used 64 × 64 pixels as the baseline, as the larger cell nuclei can be included in the image. We also evaluated the classification performance with the matrix sizes of 96 × 96 and 128 × 128 pixels. Here, the image of the captured specimen had a wide field of view, and the patch images contained areas without cells. Therefore, the image was binarized to divide it into regions with cells and background regions. If the area of cells occupied more than 10% of the area in the patch image of the instance candidate, the instance was registered as a malignant or benign bag for analysis; otherwise, it was deleted. The binarization threshold was calculated for each image using the Otsu's automatic binarization algorithm.

### Classification method

**Figure 3:**
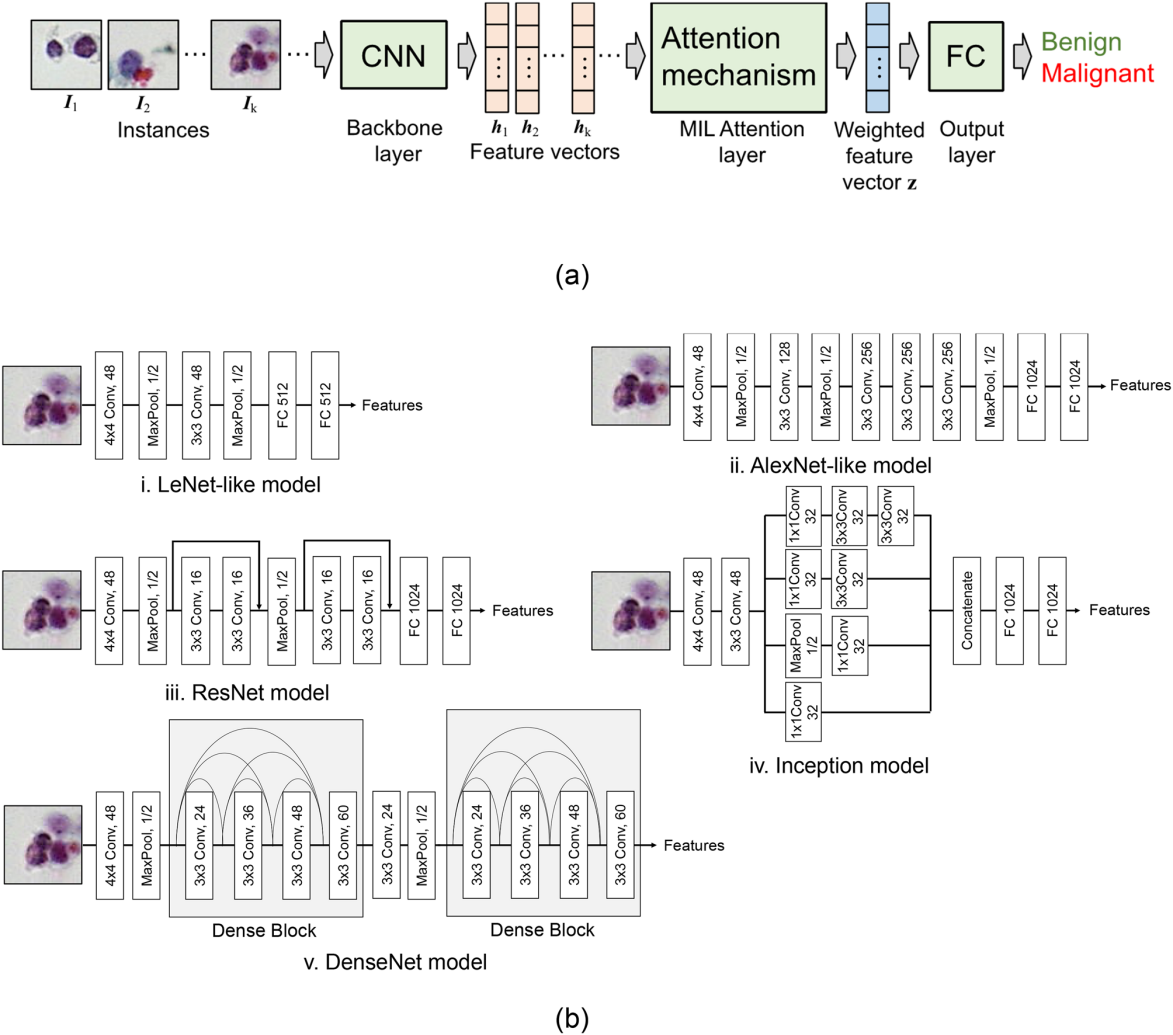
Architecture of the AD MIL. (**a**) Overall structure of the AD MIL. (**b**) CNN model for feature extraction.

The structure of the AD MIL used for classification is shown in Fig. 3^18^, and consisted of a backbone layer, an MIL attention layer, and one output layer (Fig. 3a). The backbone layer was composed of a CNN, and by providing the instance (***I***_k_) in the bag into the CNN, feature extraction was performed, and the feature vector ***h***_k_ was obtained. Here, we employed LeNet-like^19^, AlexNet-like^20^, inception architecture (Inception)^21^, networks with residual structure (ResNet)^22^, and dense connected structure (DenseNet)^23^ (Fig. 3b) as CNN in the backbone layer, and compared their classification performance. The structure of the CNN was determined empirically.

In the conventional MIL technique, the synthesized vector, which is obtained by calculating the maximum or average value of the elements of the feature vector group in the bag, was used for identification. However, the importance of an instance could not be accurately recognized with the simple calculation of the maximum or average values in the conventional method. Therefore, Ilse et al. proposed an MIL technique using an attention mechanism^18^. Attention mechanism allows the control of the neural network to pay more attention on instances that are most likely to be labeled as positive. It detects key information from a large amount of inaccurate data, which is consistent with the practical diagnosis process.

In this method, a neural network is used to calculate the attention weight, which represents the importance of an instance, and a weighted feature vector **z**, which is a weighted average of the instances, is obtained using the following equations^18^.

1 $${\varvec{z}}=\sum_{k=1}^{K}{a}_{k}{{\varvec{h}}}_{k}$$

2 $${a}_{k}=\frac{\mathrm{exp}\left\{{\mathbf{w}}^{\intercal }\mathrm{tanh}\left(\mathbf{V}{\mathbf{h}}_{{\varvec{k}}}^{\intercal }\right)\right\}}{\sum_{j=1}^{j=K}\mathrm{exp}\left\{{\mathbf{w}}^{\intercal }\mathrm{tanh}\left(\mathbf{V}{\mathbf{h}}_{{\varvec{j}}}^{\intercal }\right)\right\}}$$

where ***h***_k_ is the feature vector of the kth instance, and *a*_k_ the attention weight given for each instance and normalized so that the total value of *a*_k_ is 1 for each bag. Two vectors of **w** and **V** are the parameters for calculating the attention weight, which are determined by training the network. The weighted features **z** were then assigned to a fully connected (FC) layer with a single artificial neuron, and bag classification (benign and malignant) was performed. Here, a sigmoid function was introduced as the activation function of the artificial neuron. Therefore, AD MIL weights the instances in the bag to create a single vector that is representative of the bag, which is then used to identify whether the bag is benign or malignant. The code of the AD MIL used in this study was a modified version of the code proposed by Ilse et al.^18^, and we used a Python program developed using Keras and Tensorflow as APIs for deep learning.

### Evaluation metrics

To confirm the effectiveness of the proposed method, we evaluated its classification accuracy. All cases were used to evaluate the ability of classification between benign and malignant on a case-by-case basis using the tenfold cross-validation method. Cross-validation is a method that divides data into multiple datasets, and repeatedly trains and evaluates them to obtain classification accuracy for all data. In this study, we divided the data into 10 sets, used 9 of the 10 sets as training data, and evaluated the remaining dataset, which was repeated 10 times. In each training, 10% of the training data were used as validation data while monitoring the training error. The number of training epochs was 100, the batch size was 1, and Adam^24^ was used as the optimization algorithm. The learning rates, β1, and β2, were 0.0005, 0.9, and 0.999, respectively. We also used a computer with an Intel core i7 7800X CPU and an NVIDIA Quadro RTX 8000 GPU for training.

This method was aimed at improving the performance of MIL by using the attention mechanism. To confirm the effectiveness of the attention mechanism, we compared the classification performance with the attention mechanism disabled. Specifically, as in the conventional MIL, the feature vectors extracted from each instance were averaged, and the combined vector was calculated and used for classification.

Furthermore, to compare our method with supervised learning, we performed general supervised learning on data where all case images contained labels (benign or malignant). We prepared a CNN with an AlexNet-like structure, which was also used in the AD MIL model, with one additional FC layer, and classified it as benign or malignant using the Softmax function. Then, we trained and evaluated the prediction results for each image using the tenfold cross-validation method, as in the evaluation of the AD MIL. Based on the obtained prediction results, we calculated the confusion matrix and classification accuracy by calculating the prediction probability for each image. Note that AD MIL was evaluated on a case-by-case basis, and in actual clinical practice, if even one malignant cell is included in an image, it is considered malignant. Therefore, in the classification by supervised learning, the maximum value of the malignancy probability was obtained for all the images included in one case^13^. Based on the maximum probability, benign and malignant images were classified, and the overall classification accuracy per case was evaluated. The number of training epochs was 100 and the batch size was 16. We also employed Adam optimization algorithm with the learning rates of 0.00005, β1 of 0.9, and β2 of 0.999. The same parameters as in MIL were used, except for a batch size of 16. Then, for cases classified as malignant by AD MIL, the distribution of attention weights was determined for the instances comprising a single image, and a color attention map was obtained. This allowed us to analyze which part of the specimen was the focus.

## Results

**Figure 4:**
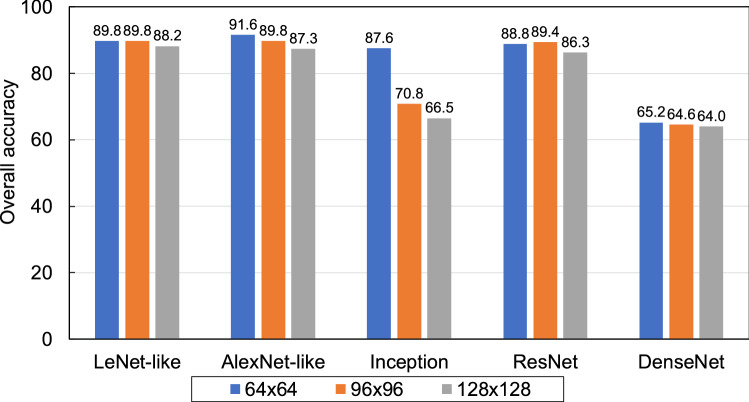
Accuracy comparison of different patch sizes.

Figure 4 shows the accuracy of the proposed method for classifying benign and malignant patches while changing the matrix size of the patch images. As a result, the best performance was obtained when the matrix size of the patch image was set to 64 × 64 pixels, which was used in subsequent analyses.

Tables 1, 2, 3, 4, 5, 6 and 7 show the confusion matrices obtained by summing up the results of classification by AD MIL while changing the CNN structure, and the results of image-based and case-based classification by supervised learning. Sensitivity, specificity, accuracy, and balanced accuracy are shown in Table 8. Here, balanced accuracy is expressed as the average of sensitivity and specificity, and is used when the number of images in the data differs between two classes. In the evaluation results, the classification by AD MIL, which adopts an AlexNet-like structure for CNN, showed the best classification accuracy.

**Table 1:** Confusion﻿ matrix of weakly supervised learning with LeNet-like model.

	Predicted: Benign	Predicted: Malignant
Actual: Benign	98	10
Actual: Malignant	23	191

**Table 2:** Confusion matrix of weakly supervised learning with AlexNet-like model.

	Predicted: Benign	Predicted: Malignant
Actual: Benign	96	12
Actual: Malignant	15	199

**Table 3:** Confusion matrix of weakly supervised learning with Inception model.

	Predicted: Benign	Predicted: Malignant
Actual: Benign	95	13
Actual: Malignant	27	187

**Table 4:** Confusion matrix of weakly supervised learning with ResNet model.

	Predicted: Benign	Predicted: Malignant
Actual: Benign	99	9
Actual: Malignant	27	187

**Table 5:** Confusion matrix of weakly supervised learning with DenseNet model.

	Predicted: Benign	Predicted: Malignant
Actual: Benign	34	74
Actual: Malignant	38	176

**Table 6:** Confusion matrix of supervised learning: image-based evaluation (AlexNet-like model).

	Predicted: Benign	Predicted: Malignant
Actual: Benign	33,542	6013
Actual: Malignant	7114	62,813

**Table 7:** Confusion matrix of Supervised learning: case-based evaluation (AlexNet-like model).

	Predicted: Benign	Predicted: Malignant
Actual: Benign	77	31
Actual: Malignant	3	211

**Table 8:** Classification results.

Learning method	CNN model	Sensitivity	Specificity	Accuracy	Balanced accuracy
Weakly supervised learning	AD MIL LeNet-like	0.893	0.907	0.898	0.900
	Conventional MIL pooling LeNet-like	0.921	0.778	0.873	0.850
	AD MIL AlexNet-like	0.930	0.889	0.916	0.910
	Conventional MIL pooling AlexNet-like	0.893	0.750	0.845	0.822
	AD MIL Inception	0.874	0.880	0.876	0.877
	Conventional MIL pooling Inception	0.897	0.528	0.773	0.713
	AD MIL ResNet	0.874	0.917	0.888	0.900
	Conventional MIL pooling ResNet	0.921	0.778	0.873	0.850
	AD MIL DenseNet	0.822	0.315	0.652	0.569
	Conventional MIL pooling DenseNet	1.000	0.000	0.665	0.500
Supervised learning: image-based evaluation	AlexNet-like	0.898	0.848	0.880	0.873
Supervised learning: case-based evaluation	AlexNet-like	0.985	0.713	0.849	0.849

**Figure 5:**
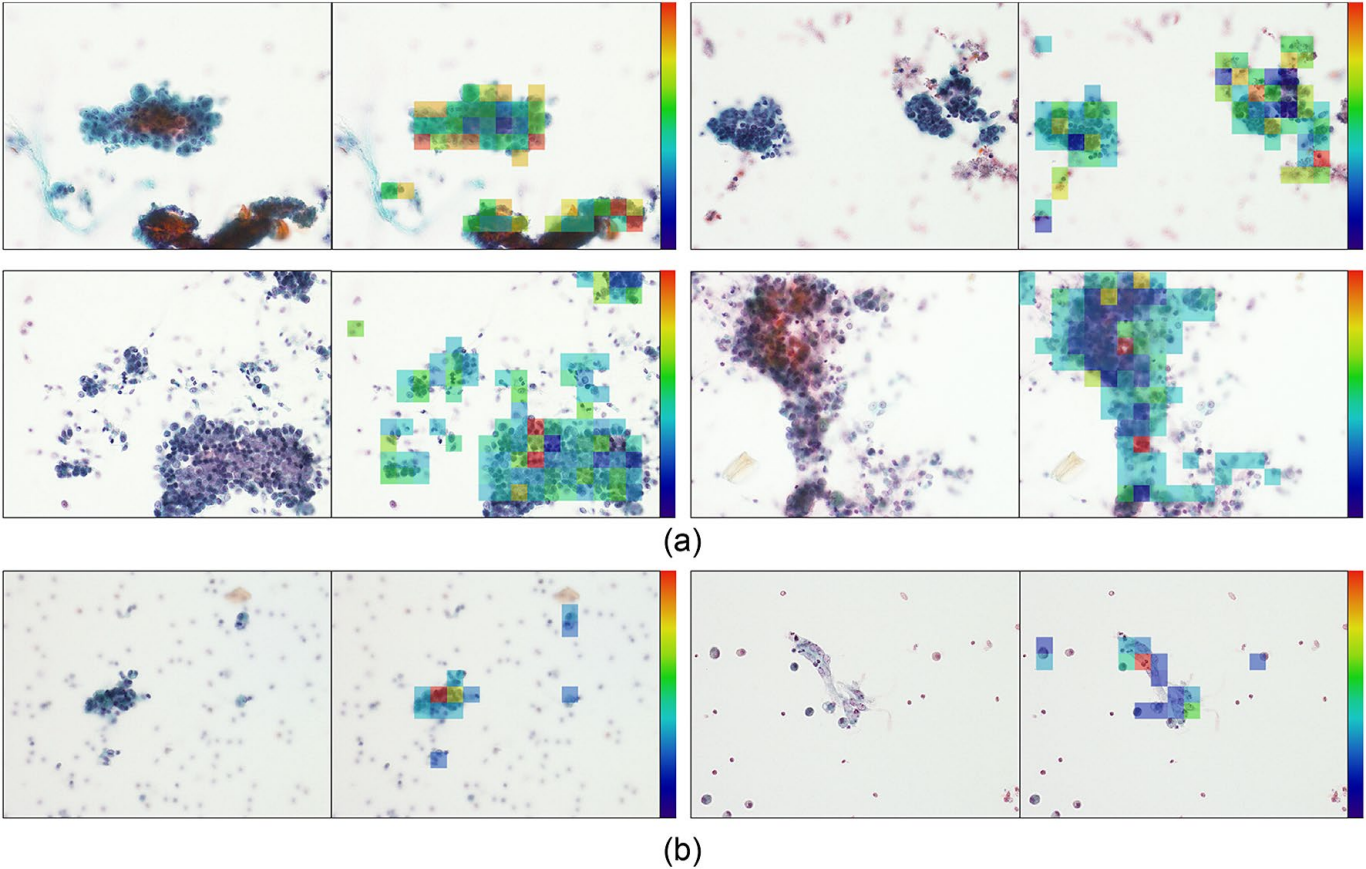
Classification result and attention maps on attention weight of benign cells. (**a**) Correctly classified benign cells. (**b**) Mis-classified benign cells.

**Figure 6:**
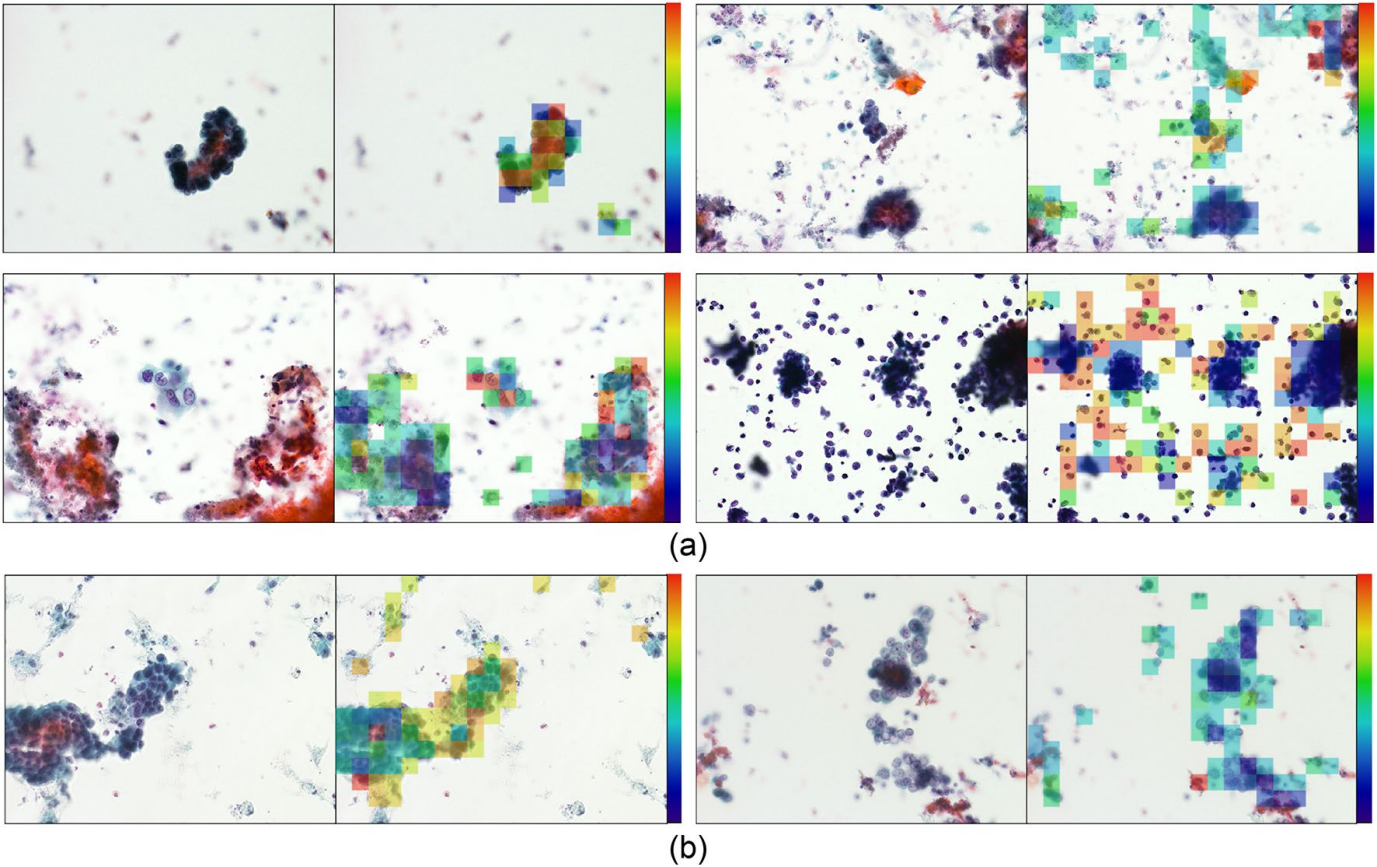
Classification result and attention maps on attention weight of malignant cells. (**a**) Correctly classified malignant cells. (**b**) Mis-classified malignant cells.

An example of the overall microscopic images of benign cases that were correctly and incorrectly classified by the AD MIL is shown in Fig. 5. In the right side of paired images are attention maps drawn by the attention weights of all instances. Similarly, the image of a malignant case is shown in Fig. 6.

## Discussion

In this study, we developed a method for the classification of benign and malignant lung cytological images using AD MIL, a weakly supervised learning method. Three CNN models for the AD MIL were compared, and the AlexNet-like model showed the best classification performance, with an accuracy of 0.916. For general image classification tasks, inception and dense model, which have complex structures, show good performance. These are mainly trained using 224 × 224 pixels as input and a large number of images. In the comparative experiments conducted in this study, the AlexNet-like model with a simple structure showed the best performance. In this study, the weights in the network are trained from the initial state to extract image features from images as small as 64 × 64 pixels. From the experimental results, it is considered that the simpler architecture performed better feature extraction than the more complex one.

Upon comparing the performance of the proposed method, with and without the attention mechanism, the results of the method with the attention mechanism (CNN: AlexNet-like model) showed up to 9% higher classification performance with balanced accuracy, indicating that the attention mechanism is effective for cell classification. The results showed that the attention mechanism was effective in classifying cells.

As a supervised learning method, image classification was performed with the AlexNet-like CNN model used in AD MIL, and the results show that AD MIL was slightly better than supervised learning. In the attention maps presented in Figs. 5 and 6, the focus of attention is slightly different between malignant and benign cases. In malignant cases, the cells that are strongly suspected to be malignant are at a high attention level (yellow or red). On the contrary, in benign cases, attention tended to be paid to the whole area or to the periphery of the cells. Benign cells have a relatively uniform shape of nuclei and may show striae around the cytoplasm; it is believed that AD MIL has been trained to classify the abovementioned differences. Even when a benign image is incorrectly classified as malignant, the cytotechnologist or cytopathologist only needs to confirm the area focused on by AD MIL, which may shorten the diagnosis time.

The best matrix size for the instance (patch image) given to AD MIL was 64 × 64 pixels. This is thought to be the size of the field of view where a single cell fits in the microscopic image and attention to individual cells is engaged.

Both malignant and benign cases contain a variety of cell types. For malignant cells, as mentioned above, the shape of the nucleus and cytoplasm, as well as the structure, arrangement, and color of the inside of the nucleus, differ from those of benign cells. In addition, there are three subcategories of lung cancer, each of which has different characteristics and incidence rates. The breakdown of the types of lung cancer used in this study is 58% adenocarcinoma, 24% squamous cell carcinoma, and 18% small cell carcinoma; these percentages reflect the actual percentage of patients. Because the proposed method could be used for correct classification of benign and malignant cells among these case groups, it appears to have identified the characteristics contained in the three types of malignant cells and discriminated malignant cells from benign cells.

In our previous studies, classification of benign and malignant lung cytological images was performed with nearly 90% accuracy. If experts such as cytolopathologists accurately label individual cytological images as benign or malignant, good classification performance can be achieved. However, a cytological specimen obtained from a single patient contains a large number of cells, and it is extremely difficult to designate the benign and malignant properties of each cell. In addition, when experts evaluate these specimens, they do not classify them as benign or malignant on a cell-by-cell basis, but rather make a comprehensive diagnosis, and thus the classification methods used by experts and supervised learning are different. Rather than assigning a correct label to each cell, our approach was to divide a large number of cell images obtained from a single patient into small patch images (instances) and classify them comprehensively on a bag-by-bag basis. In addition, CNN was used to obtain advanced features from the images, and an attention mechanism was used to select and evaluate the images of interest. Therefore, this method is closer to the clinical diagnosis procedure than conventional supervised learning: it can classify a large number of images with high accuracy without the need for complicated work by experts.

The field of view in this study was relatively small because the images were collected by a camera attached to a microscope; this method may be more useful when using whole slide images containing a large number of cells.

A limitation of this study was that the specimens were collected at a single institution, and the number of cases was small. In the future, it will be necessary to collect specimens from other institutions and construct a more robust classification model. Thus, the practical effectiveness should be evaluated via external validation. In this paper, we show results of various experiments on AD MIL, and processed them by supervised learning method using the same patch images as a comparison. Considering various learning models have been proposed^25,26^, it is necessary to compare the results with those, in the future.

In cytological diagnosis, classification of cancer type is required after the identification of the benign and malignant status. We have proposed a method for automatic classification of the histological types of lung cancer based on supervised learning^27^. In the future, we plan to develop a tissue type classification method based on a weakly supervised learning method.

## Conclusion

In this study, we developed a method for classification of cytological images as benign or malignant using AD MIL, and compared its ability with classification by supervised learning. We show that weakly supervised learning with AD MIL was able to reach the acceptable accuracy obtained with supervised learning and visualize the regions that contributed to the decision by the attention mechanism. We conclude that our method automatically classifies cytological images more easily and accurately than methods based on supervised learning.
